# Sociocultural and interpersonal influences on latina women’s beliefs, attitudes, and experiences with gestational weight gain

**DOI:** 10.1371/journal.pone.0219371

**Published:** 2019-07-24

**Authors:** Ana Cristina Lindsay, Márcia Maria Tavares Machado, Sherrie F. Wallington, Mary L. Greaney

**Affiliations:** 1 Department of Exercise and Health Sciences, University of Massachusetts–Boston, Boston, United States of America; 2 Department of Community Health–School of Medicine, Federal University of Ceará, Fortaleza, Brazil; 3 School of Nursing, George Washington University, Washington, DC, United States of America; 4 Health Studies & Department of Kinesiology, University of Rhode Island, Kingston, United States of America; Chinese Academy of Medical Sciences and Peking Union Medical College, CHINA

## Abstract

Latinos are the largest and fastest-growing minority group in the U.S., and Latina women represent the largest portion of minority births, having the highest birth rate in the U.S. for over 20 years. In addition, Latina women are at increased risk of entering pregnancy being overweight or having obesity and gaining excess gestational weight. Excess gestational weight gain (GWG) has short- and long-term adverse health outcomes for the woman and her child. Although culturally tailored interventions show promise toward promoting healthy GWG among Latina women, findings from current interventions have had mixed results, suggesting the need for further tailoring to meet the needs of this heterogeneous population group. This qualitative study was designed to explore first-time pregnant, low-income Latina women’s beliefs, attitudes, and experiences with GWG. The study employed qualitative research using semi-structured interviews conducted with 23 first-time pregnant Latina women between 22 and 36 weeks of gestation. Interviews were conducted by trained bilingual staff, transcribed verbatim, and analyzed using thematic analysis. Results showed that participants were uncertain if their GWG was within a healthy range. Although the majority of participants knew that GWG should be limited, they were not sure what the amount should be. In addition, the majority of participants reported attitudes of acceptance of and resignation to excessive GWG as being part of pregnancy. Several women appeared to believe that they did not have control over their weight gain during pregnancy. Moreover, analysis identified that sociocultural and interpersonal factors such as social support influence the beliefs, attitudes, and experiences with GWG of the low-income, majority immigrant Latina women who participated in this study. Study findings can be used to further tailor prenatal care practices and interventions aimed at altering modifiable risk factors associated with excess GWG among Latinas. Future interventions designed for low-income, immigrant Latina women that consider sociocultural influences on women’s beliefs and attitudes related to GWG, as well as the influence of social support networks on women’s health behaviors during pregnancy, will likely be more effective in preventing excessive GWG.

## Introduction

Latinos are the largest and fastest-growing minority group in the U.S. [1], and Latina women represent the largest portion of minority births, having the highest birth rate in the U.S. for over 20 years [2]. In addition, evidence shows Latina women are at increased risk of entering pregnancy overweight or obese [3].

Excessive gestational weight gain (GWG) is a risk factor for obesity that has short- and long-term adverse health outcomes for the woman (e.g., gestational diabetes, postpartum weight retention, risk of obesity, etc.) and her child (e.g., birth weight, risk of obesity) [3,4]. Moreover, evidence suggests that excessive GWG contributes to disparities in obesity among Latino children [5,6], which first emerge in the transmission of intergenerational risk factors for obesity in the earliest life stage (i.e., pregnancy through 24 months of age) [5,6].

A range of factors influence excessive GWG, including maternal sociodemographic characteristics [5,6], medical care [7], gestational conditions [3,4], pre-pregnancy body mass index (BMI) [7,8], maternal factors (such as maternal age) [9], parity [8], and maternal behaviors such as smoking [9], dietary intake [9], and physical activity [10]. Additionally, a growing body of literature indicates that structural inequalities (e.g., access to health care, health insurance coverage, physical and financial resources, material hardships, etc.) influence low-income minority women’s risk of excessive GWG [3,4,10,11]. Research also suggests that women’s knowledge, beliefs, and attitudes about GWG are influenced by sociocultural factors (e.g., income, education, social support and social networks, race and ethnicity, language, and acculturation) [12,13].

Despite an understanding of a range of factors associated with excessive GWG, interventions to prevent excessive GWG in the general population [14,15] and among Latina women [16–19] have had mixed results. A systematic review and meta-analysis of antenatal dietary and lifestyle interventions (n = 9) including studies involving multiracial women to reduce excessive GWG concluded that the number and quality of published trials was insufficient to allow evidence-based recommendations for clinical practice to be developed [8]. Similarly, another systematic review of behavioral interventions for weight management in pregnancy including studies involving multi-racial samples and including quantitative and qualitative studies (five controlled trials and eight qualitative studies), found no significant difference in GWG among participants in the intervention group compared with the control group [15].

In contrast, a more recent systematic review and meta-analysis including studies involving multiracial women showed positive intervention results and found that most dietary and lifestyle interventions in pregnancy reduce GWG without having an adverse effect on the risk of babies being small for gestational age [14]. Overall, findings of this systematic review and meta-analysis showed that interventions focusing on healthy eating had a larger effect size in limiting GWG than interventions that addressed both healthy eating and physical activity [14]. On the other hand, interventions that focused solely on physical activity did not show a significant pooled effect [14].

Furthermore, other studies conducted among multiracial pregnant women including Latinas have found a lack of awareness among women who are overweight or have obesity regarding excessive GWG and have identified barriers (e.g., getting time off from work; location of services offered; intensity of intervention, which limited compliance) that impede the use of resources to address excessive GWG [13,14,16,17].

Moreover, preliminary findings of a recent feasibility study of a randomized controlled trial of a lifestyle intervention with Latina women, *Proyecto Mamá*, suggested that the intervention helped mitigate a decline in vigorous physical activity over the course of pregnancy among Hispanic women who were overweight or had obesity [16–18]. Although the study had a relatively small sample size (n = 68), it demonstrated that strategies for helping pregnant Latina (Hispanic) women overcome barriers to physical activity participation and to attending group sessions are needed for such interventions to be successful [18].

Similarly, another recent trial designed to test the feasibility and efficacy of a prenatal behavioral intervention in a sample of low-income, predominantly Latina women showed initial positive results [19]. Compared to usual care, fewer healthy-weight women in the intervention arm exceeded recommendations for GWG (47.1% usual care vs. 6.7% intervention; absolute difference 40.4%). Nonetheless, frequently changing work schedules made it challenging to ensure that women attended a sufficient number of group sessions or developed behavior change skills through other modalities [19].

These findings combined suggest that a greater understanding of low-income women including Latinas’ beliefs, attitudes, and experiences with GWG is needed to further identify and address barriers to participation and compliance to interventions designed to promote healthy GWG. The present qualitative study further explored these topics.

## Methods

The present study (n = 23) was part of a larger ongoing, community-based, mixed-methods exploratory research study (n = 150) [20]. The overall goal of the larger study is to explore and identify factors associated with risk of obesity in the first 1,000 days of life in multi-ethnic Hispanic and Brazilian families living in Massachusetts (MA) and Rhode Island (RI). We used the socioecological model (SEM) as a framework for this study [21]. The SEM considers intrapersonal, interpersonal, environmental, organizational, and policy influences on health and can be used to identify factors that are potentially amenable to intervention and modification [21]. In addition, we also considered the social contextual model (SCM), an adaptation of the SEM developed by Sorensen et al. [22], which integrates social class and culture to the levels posited by the SEM. The SCM emphasizes the expression of cultural pathways that may explicitly inform the design of health promotion interventions [22].

This study was conducted in accordance with guidelines for the Consolidated Criteria for Reporting Qualitative Research (COREQ) [23]. Using an exploratory descriptive qualitative methodology [24], individual, in-depth interviews were conducted with first-time pregnant immigrant Latina women to explore (a) beliefs about, attitudes toward, and experiences with GWG during pregnancy; and (b) perspectives about and current experiences with patient-provider communication related to GWG during pregnancy.

Qualitative research methods allow researchers and participants to discover and explore topic areas without predetermined quantitative questions [25]. In-depth, semi-structured interviews were the qualitative research method chosen for this study because they allow for the collection of valuable information in diverse cultural settings [25]. Moreover, individual interviews were preferred over focus groups given the challenge of assembling a group of pregnant women, most of whom, were in their third trimesters. This study received ethical approval from the University of Massachusetts–Boston Ethics Board (IRB#2013132).

### Participants

Women were eligible to participate in the current study if they (a) self-identified as Latina (either Hispanic or Brazilian); (b) were expecting their first child (a single birth), and were at 22–36 weeks gestation; (c) were 18 years of age or older; (d) were participating in or eligible for participation in the Special Supplemental Nutrition Program for Women, Infants and Children (WIC); (e) lived in MA and RI; (f) resided in the U.S. for at least 12 months; and (g) provided signed informed consent.

A convenience sample of participants was recruited through flyers that described the study (e.g., purpose of study, researchers involved, funding, extent of participation, etc.) and included a telephone number. Flyers were posted at community-based social and health organization and churches between April 2016 and February 2017. Women who called the posted telephone number were screened via telephone by study staff. After determining study eligibility, study staff then scheduled interviews with women who met the eligibility requirement. A total of 26 interested participants contacted the research staff via telephone. One of the 26 participants did not meet study eligibility criteria, and two eligible participants did not complete their scheduled interview despite follow-up calls and were classified as dropping out of the study.

### Data collection

Native Spanish (AH) and Portuguese (ACL, GDA) speakers trained in qualitative research methods conducted all interviews in Spanish or Portuguese from the same semi-structured interview guide, which included open-ended questions and probes (see Table 1). Interviews were held at public locations (library, community agency, church) convenient for participants or, if requested, at the participant’s house. Trained bilingual (Portuguese and English or Spanish and English) research assistants took notes during all interviews.

**Table 1:** Interview questions on Latina women’s beliefs about, attitudes toward, and experiences with gestational weight gain during their first pregnancy.

**Knowledge**	Do you know how much weight you are supposed to gain in pregnancy? How do you know? Have you received, heard, or been told of any specific recommendations about weight gain during pregnancy? From whom did you hear this information? *Probe*: *family*, *partner*, *friends*, *and health care provider?*
**Beliefs**	How much weight do you think you should gain? What does gaining too much weight in pregnancy mean to you? Why do some women gain too much weight in pregnancy? Does family/cultural pressure influence your eating habits more than what your doctor said? Have you been encouraged to eat for two? Are you afraid you are not eating enough and ‘starving’ baby? What have you heard about that women should eat during pregnancy? Where did this information come from? Are you following any of this information? Overall, are you eating differently from when you were not pregnant and if so how? *Probes*: *(eating more or eating less*, *satisfying cravings*, *consuming more junk food/fast food*, *consuming more or less of vegetables*, *meats*, *grains*, *dairy*, *fruits*, *dealing with nausea*, *lack of appetite etc*.*)* Is it important to you to get back to your pre-pregnancy weight after your baby is born? *Probe*: *self-image*, *pressure from partner or family*, *health concerns*, *etc*.
**Attitudes**	How do you feel about weight gain during this pregnancy? *Explain*. *Probe*: *self-image (fat*, *ugly*, *awkward*, *beautiful)*, *attitudes of others (uninvited touching*, *staring*, *comments*, *unsolicited advice)*, *and concerns about own health vs*. *baby’s health*. Are there benefits from gaining too much weight in pregnancy? *Probe*: *For mothers? For babies? What are they?* Are there problems from gaining too much weight in pregnancy? *Probe*: *For mothers? For babies? What are they?* Is it possible to control the amount of weight you gain in pregnancy? Why or why not? Who influences what you think about your pregnancy weight gain? *Probe*: *family*, *partner*, *friends*, *and health care provider*, *information on magazines and books*, *information on the internet?* Whose advice or opinion about weight gain during pregnancy do you most value or trust? *Probe*: *family*, *partner*, *friends*, *and health care provider*, *information on magazines and books*, *information on the internet?*
**Experiences**	How do you feel about your pre-pregnancy weight and potential weight gain during this pregnancy? What has been your experience with weight gain during this pregnancy? Did you ask your doctor about weight gain or did s/he bring it up? How much weight you were advised to gain? If s/he did bring it up initially, do you discuss weight gain at each visit? If you visit a practice where you see different doctors, have you received conflicting information? **Now, let’s talk about after the baby is born.** What has your doctor told you about losing weight after your baby is born? Is it important to you to get back to your pre-pregnancy weight after your baby is born? Explain. *Probes*: *self-image*, *pressure from partner or family*, *health concerns etc*. Do you know of women who gained a lot of weight during pregnancy and were not able to take it off afterwards? How would you feel about that if it were to happen to you? What are you planning to do to help yourself return to your desired weight? Probe for dieting, increasing physical activity, joining a gym, etc.

Interviews took place between June 2016 and March 2017 and lasted approximately 45 minutes. Prior to the start of all interviews, participants were given written information—either in Spanish or Portuguese—about the study. All interviews were audio-recorded after participants provided signed and oral informed consent.

The pilot-tested interview guide explored three primary domains of inquiry: (1) participants’ beliefs about and attitudes toward GWG; (2) participants’ experiences with GWG; and (3) patient-provider communication and advice related to GWG and PA during pregnancy. This paper focuses on the first two domains of inquiry (i.e., beliefs and attitudes toward GWG and experiences with GWG). Results of the third domain of inquiry are presented elsewhere [20].

All participants completed a brief, self-administered survey after the interview ended. The survey assessed participants’ socio-demographics (education, marital status, country of origin, length of time living in the U.S., etc.), self-reported perceived weight status prior to start of pregnancy, and level of acculturation via the Short Acculturation Scale for Hispanics (SASH), [26]. The SASH is a 12-item measuring scale validated for use in Latino groups such as, Mexican Americans, Cuban Americans, Puerto Ricans, Dominicans, and Central and South Americans that assesses language use, media use, and ethnic social relations [26]. An acculturation score was computed by averaging across 12 items as measured on a scale of one to five (1 = least acculturated, 5 = fully acculturated) [26]. Participants received a $25 gift card at the end of the interview for their participation.

### Data analysis

The interviewers and research assistant met for about 20 minutes at the end of each interview to review new and recurring themes. All themes were entered into a grid that was used to monitor emerging themes and to determine when data saturation occurred. Professional transcriptionists and native Spanish and Portuguese-Brazilian speakers transcribed all audio recordings verbatim without identifiers. Using the SEM and SCM as guiding frameworks [21,22], Spanish and Portuguese transcripts were analyzed separately using thematic analysis, an iterative process of coding the data in phases to create meaningful patterns by two experienced and trained (PhD) qualitative researchers, both native Portuguese (Brazilian) speakers (ACL, MMTM) who are also language proficient in Spanish. Each researcher read several transcripts numerous times to become familiar with the content and generate initial codes [27]. The researchers then manually coded transcripts independently but met regularly to discuss coding and to identify and resolve disagreements in coding [27]. The coded texts describing similar ideas were grouped and sorted to identify emergent themes and subthemes. Finally, salient text passages were extracted and translated into English by a multilingual professional translator using forward-backward process to ensure semantic equivalency. The text passages were used as illustrative quotes for the emergent themes. Descriptive statistics and frequencies were calculated for data collected in the socio-demographic survey using Microsoft Excel 2008.

## Results

A total of 23 individual semi-structured interviews were conducted with a diverse sample of Latina pregnant women (12 multi-ethnic Hispanics and 11 Brazilians). Participants’ characteristics are presented in Table 2. Women’s (n = 23) ages ranged from 22 to 35 (mean = 24.1, SD = 2.3) years of age. Nearly all participants (n = 22, 96%) were foreign-born and had lived in the U.S. for an average of 8.2 years (SD = 2.4). No participants spoke English as their primary language, and participants had a mean acculturation score of 1.62 (SD = 0.37), indicating that they identified more closely with Latino culture than with U.S. culture. About 83% of women were married. About 44% had completed high school (n = 10) or obtained a general education degree (GED; n = 4; 17.3%), and the majority (n = 19; 87.3%) reported family annual income of ≥ US $20,000 and < US $40,000/year. Last, a little over half (*n* = 12; 53.2%) of the participants reported being a healthy weight prior at the start of pregnancy, while about 44% (*n* = 10) reported being overweight, and only one participant self-reported having obesity prior to the start of pregnancy.

**Table 2:** Sociodemographic and acculturation characteristics and self-reported weight status of study participants (n = 23).

	Mean + SD	n (%)
**Age** (yrs.)	24 + 2.3	
**Race**		
Brazilian		11 (47.8)
Hispanic		12 (53.2)
**% Foreign born**		22 (95.6)
**Country of origin**		
Brazil		10 (45.5)
Dominican Republic		4 (18.2)
Puerto Rico (United States)		4 (18.2)
Colombia		2 (9.1)
El Salvador		1 (4.5)
Guatemala		1 (4.5)
Years in the United States*	8.2 + 2.4	
**Predominant language spoken at home**		
Spanish		12 (53.2)
Portuguese		11 (47.8)
**Marin scale acculturation score**	1.62 + 0.37	
**Marital status**		
Married		19 (82.7)
Single		4 (17.3)
**Educational level**		
Less than high school		6 (26.1)
High school degree		10 (43.6)
General Education Degree (GED)		4 (17.3)
Some college or more		3 (13.0)
**Household annual income**		
> $20K/year < $40,000		19 (82.7)
< $20K/year		4 (17.3)
**Percent employed**		17 (73.9)
**Self-reported BMI (kg/ m**^**2**^**) prior to pregnancy**		
Healthy weight		12 (53.2)
Overweight		10 (43.6)
Obese		1 (4.4)

* Limited to foreign-born women.

Thematic content analysis identified 13 emergent themes within two examined domains: (1) women’s beliefs about and attitudes toward GWG (eight themes), and (2) women’s experiences with GWG (six themes). Themes were organized using the SEM and SCM [41,42]. Emergent themes are presented in Table 3, with representative quotes translated to English to illustrate findings.

**Table 3:** Interview themes and supporting quotes from Latina women (n = 23) regarding their beliefs, attitudes, and experiences with gestational weight gain.

Themes	Representative Quotes	
**Domain 1: Women’s Beliefs and Attitudes Toward Gestational Weight Gain**		
**Intrapersonal Influences**		
**Theme 1: Sense of acceptance**	*“I see my body changing and at times it can be very upsetting … but it’s like I can’t do much about it*. *I have to accept that gaining weight is part of being pregnant*.*”(Participant #21*, *Brazilian*, *self-reported as healthy weight before pregnancy)*. *“It’s not that I want to gain a lot of weight*, *but gaining weight is part of being pregnant*. *So*, *I just try not to get too preoccupied and enjoy being pregnant*.*” (Participant #1*, *Salvadoran*, *self-reported as overweight before pregnancy)*	
**Theme 2: Uncertainty about how much weight should be gained**	*“I think I am gaining the right amount because it’s not like the doctor has mentioned she is concerned or anything*. *She [doctor] never really brought up*, *so I think I am Ok*.*” (Participant #15*, *Brazilian*, *self-reported as healthy weight before pregnancy)* *“I am not really sure if I am gaining the right amount*. *I think I am because my doctor never really told me that I wasn’t*. *She always looks at the charts and says that everything looks good*. *I think she would have mentioned it if it wasn’t right*.*” (Participant #12*, *Puerto Rican*, *self-reported as overweight before pregnancy)*.	
**Theme 3: Knowledge and information sources about healthy gestational weight gain**	*“I think it’s about 30 pounds*, *but I have read that it also depends on your weight prior to pregnancy*, *and if you have any other health issues*.*”* (Participant *#16*, *Brazilian*, *self-reported as healthy weight before pregnancy)* *“I think it’s between 35–40 pounds*. *But*, *I read in a magazine that most of the weight should happen after the first trimester*.*” (Participant #6*, *Puerto Rican*, *self-reported as overweight before pregnancy)* *“I get a pregnancy magazine every month*, *and it always has information on weight gain and how to manage weight gain during pregnancy*. *So*, *a lot of what I know is from what I have read*.*” (Participant #8*, *Colombian*, *self-reported as healthy weight before pregnancy)* *“I get a lot of information on the Internet*. *At the start of my pregnancy*, *I signed up for a website called BabyCenter*. *Every week*, *I receive messages with lots of information about what to expect and what’s happening with the baby during that stage*.*” (Participant #17*, *Brazilian*, *self-reported as healthy weight before pregnancy)* *“I was above a good [healthy] weight when I started my pregnancy*, *so during one of my first visits my doctor talked to me about weight gain during pregnancy*.*” (Participant #7*, *Puerto Rican*, *self-reported as obese before pregnancy)*	
**Theme 4: Women who self-report healthy weight prior to pregnancy appear to be more concerned about GWG**	“*My body feels so different … I feel so big*, *but people keep telling me that I am Ok*. *I was really thin prior to pregnancy*, *so I feel like I’ve gained a lot [weight]*. *I hope it’s all baby weight*, *and that it will come off once the baby is born*. *I really hope so*.*” (Participant #4*, *Dominican*, *self-reported as healthy weight before pregnancy)* *“It’s not that I was skinny before getting pregnant*. *I was already a good size*, *so I knew that I would be gaining more weight during pregnancy and I wasn’t really concerned about my weight gain as long as I was feeling well and the baby was fine …” (Participant #7*, *Puerto Rican*, *self-reported as obese before pregnancy)*	
**Theme 5: Sense of lack of control over and limited concerns about excess gestational weight gain as long as baby is fine**	*“At the beginning of my third trimester*, *I start getting concerned that I was gaining too much weight too fast*. *So*, *I started to pay more attention to what and how much I ate*, *but it didn’t really make any big difference*. *I still kept putting on a lot of weight*.*” (Participant #11*, *Guatemalan*, *self-reported as overweight before pregnancy)* *“Throughout my pregnancy I have tried as much as possible to watch what and how much I eat*, *but no matter what I do*, *I keep gaining a lot of weight … I feel it’s beyond my control*.*” (Participant #14*, *Brazilian*, *self-reported as healthy weight before pregnancy)* *“It’s not that I don’t worry about my weight*, *but like I can’t be constantly obsessing about it*. *I feel like*, *I need to focus on the positive*, *like the baby being fine*. *So*, *I am constantly telling myself*, *as long as everything is fine with the baby I will be fine … and eventually*, *the weight will come off*!*” (Participant #6*, *Puerto Rican*, *self-reported as overweight before pregnancy)*	
**Interpersonal Influences**		
**Theme 6: Freedom to eat**	*“When you are pregnant*, *you feel like you can eat as much as you want without feeling bad about it … It’s a time that there is no shaming for eating*.*” (Participant #7*, *Dominican*, *self-reported as healthy weight before pregnancy)* *“You feel supported about eating more than usual … It’s like people are supportive because you are pregnant*, *and you are eating for two*.*” (Participant #3*, *Colombian*, *self-reported as healthy weight before pregnancy)* *“When I say*, *“I can’t believe I am hungry again*!*” my friends and relatives say—you are pregnant*! *And everyone is like*, *don’t worry*, *enjoy it while you can*, *you are pregnant*!*” (Participant #14*, *Brazilian*, *self-reported as healthy weight before pregnancy)*	
**Theme 7: Sociocultural beliefs about pregnancy and food**	*“When you are pregnant it’s like there are all these beliefs that pregnant women want to eat all the time and everybody is always thinking and offering you food*. *I go to my in-laws and my mother-in-law is always making me special dishes*, *asking me what I feel like eating … It’s like people think pregnant women and they think food*.*” (Participant #18*, *Brazilian*, *self-reported as healthy weight before pregnancy)* *“My husband knows I love sweets*. *So*, *since I got pregnancy*, *he is always buying all sorts of sweets*, *cakes*, *ice cream … I tell him*, *please don’t buy all these treats because if we have treats at home*, *I will be eating them all the time … but he keeps buying and says “Ah*, *don’t worry too much*. *Enjoy it*, *you are pregnant*.*”(Participant #13*, *Brazilian*, *self-reported as healthy weight before pregnancy)* *“People believe [Hispanic culture] that an expectant woman’s cravings must be satisfied otherwise all sorts of things may happen to the expectant woman or the baby … there are all sorts of traditional beliefs and people really follow them*.*” (Participant # 11*, *Guatemalan*, *self-reported overweight before pregnancy)*	
**Domain 2: Women’s Experiences with Gestational Weight Gain**		
**Intrapersonal Influences**		
**Theme 1: Weight gain is gradual and not initially noticed**		*“It was not until I was about 31 weeks pregnant that when I stepped on the scale and the nurse was like*, *wow*, *you gained quite a bit since your last visit* .* *.* *. *and told me to make sure I was eating healthy foods*, *and avoiding junk food*. *But until that point*, *no one [health care providers] had said anything about my weight*.*”(Participant #10*, *Puerto Rican*, *self-reported as overweight before pregnancy)*
**Theme 2: Weight gain is a source of concern for some**		*“My weight and weight gain is something I think about every day*, *many times*. *You think about when you are getting dressed*, *you think about when look yourself in the mirror*, *and you think about when people look at you*. *It’s like something you can’t get away from … and it’s even more so when you are pregnant*. *It’s all about the weight*!*” (Participant #9*, *Dominican*, *self-reported as overweight before pregnancy)*
**Theme 3: Postpartum weight loss has not yet been discussed**		*“My doctor has not really talked to me about loosing weight after the baby is born*. *It’s like she [doctor] hasn’t mentioned anything*. *She [doctor] probably will talk about it after childbirth* .* *.* *. *Like*, *I think the focus now is on having a safe delivery and a healthy baby … I know that’s all that’s in my mind*!*” (Participant #1*,*Salvadoran*, *self-reported as overweight before pregnancy)*
**Interpersonal Influences**		
**Theme 4: Weight worries can wait**		*“I feel I have gained a lot of weight*, *especially in the past month*. *At my last visit I had gained 6 ½ pounds in a month*. *I asked the doctor and she said that usually pregnant women gain more weight fast at the end of pregnancy*. *She [doctor] told me all looked fine with me*, *and the baby*. *So*, *I guess like*, *I have to wait and worry about losing the weight after the baby is born* .* *.* *. *like there isn’t really much I can do now*. *As long as the baby is healthy*, *I am happy*!*” (Participant #9*, *Dominican*, *self-reported as overweight before pregnancy)* *"I was always thin and never really had any weight problems before [pregnancy]*. *So*, *I hope I can get back to my body after the baby is born*. *I think I should not worry too much about it now … that’s what everybody keeps telling me because like*, *there are days that’s easy to feel down and get depressed about it*.*” (Participant #21*, *Brazilian*, *self-reported as healthy weight before pregnancy)*
**Theme 5: Shared experience with and support from social networks**		*“Like my sister*, *when she was pregnant she really put on a lot of weight*. *It took her about a year after the baby was born to get back to a good weight*, *but she worked hard on it and like*, *she lost all the baby fat*. *So*, *I am hoping like I can also lose weight after the baby is born*.*” (Participant #20*, *Brazilian*, *self-reported as healthy weight before pregnancy)*
**Theme 6: Realization of weight gain**		*“Like me*, *I am in my 32 week and like you get towards the end and you just starting to feel huge*. *And like your body feels so different from before*, *you just keep wanting to have the baby and get back to the way your body was prior to being pregnant*, *but you are not really there yet … I just can’t wait to hold my baby and see that he’s healthy… so*, *you forget about weight and focus on the baby*.*” (Participant #4*, *Dominican*, *self-reported as healthy weight before pregnancy)*

### Domain 1: Women’s beliefs and attitudes toward gestational weight gain

#### Intrapersonal influences

Theme 1: Sense of acceptance: “Weight gain is part of pregnancy”

Nearly all participants (n = 21) viewed weight gain as part of pregnancy and accepted that their body was changing (e.g., weight, shape) due to pregnancy. Women were tolerant of weight gain even though many participants (n = 18) believed that weight gain during pregnancy might exceed recommendations or go beyond what they perceived as being the “right amount.”

Theme 2: Uncertainty about how much weight should be gained: “You hope that you are gaining the right amount”

Although most women (n = 19) believed that there was a right amount of weight that should be gained during pregnancy, the majority was not really sure what this amount was. In addition, more than half of the women (n = 15) were not certain if they were on track to gain the right amount of weight or if their GWG was within a healthy range.

Theme 3: Knowledge and information sources about healthy gestational weight gain: “I think it’s about 30 pounds, but it depends on your weight prior to pregnancy”

When asked what the right amount of weight to gain during pregnancy was, women appeared unsure. About half of the participants (n = 12) reported that women should gain between 25 and 50 pounds, with about one-third of the participants (n = 9) mentioning that it depended on weight prior to pregnancy. In addition, when further queried about how they knew the healthy range for GWG, the majority of women (n = 20) reported that they gathered this information from printed sources (books and pamphlets obtained during health care visits) and the Internet (pregnancy websites and Google searches). Only a few participants (n = 3) reported receiving information from health care professionals such as physicians and WIC staff.

Theme 4: Women who self-report healthy weight prior to pregnancy appear to be more concerned about GWG:” I was really thin prior to pregnancy, so I feel like I’ve gained a lot [weight]”

About half of the participants (n = 12) mentioned being concerned about excessive GWG and about losing the weight after having their baby. Women who self-reported being a healthy weight (n = 10 out of 12) prior to pregnancy appeared most concerned about this.

Theme 5: Sense of lack of control over and limited concerns about excessive gestational weight gain as long as baby is fine: “I feel like, I need to focus on the positive, like the baby being fine”

A few women (n = 6) reported feeling that they did not have complete control over their GWG. Moreover, several participants (n = 10) reported that they were not overly concerned about their GWG as long as everything was fine with the baby.

#### Interpersonal influences

Theme 6: Freedom to eat: “I know that I am not really eating for two, but pregnancy is a unique time”

Most participants (n = 19) referred to pregnancy as being a unique time, and several (n = 15) reported that they felt free from the shame of eating too much (n = 4). This freedom to eat was expressed by about two-thirds of the participants (n = 15), who felt they could eat as much as they wanted during pregnancy without feeling guilty or ashamed. Moreover, participants’ views on pregnancy being a period of freedom to eat as much as one wants appeared to be reinforced by their friends and family members.

Theme 7: Sociocultural beliefs about pregnancy and food: “There is all the pampering around food”

The majority of participants (n = 18) spoke of social and cultural beliefs about pregnancy that promoted a culture of pampering pregnant women with food. Additionally, about two-thirds of the women (n = 16) reported that their husbands and close family members would frequently pamper or indulge them with food. Furthermore, about one-third of participants (n = 7) spoke of social and cultural acceptance in the Hispanic and Brazilian culture of consumption of unhealthy foods or cravings during pregnancy that created an environment full of temptations. Furthermore, several participants (n = 13) reported that their beliefs that greater GWG is associated with healthier babies were shared and reinforced by close friends and family members.

### Domain 2: Women’s experiences with gestational weight gain

#### Intrapersonal influences

Theme 1: Weight gain is gradual and not initially noticed: “You start putting on weight and you don’t think much about it until you face the scale”

Nearly all participants (n = 19) mentioned that their weight gain was gradual during their pregnancy and that they did not initially notice the amount of weight gained. They spoke of realizing only late in their pregnancy that their gestational weight gain was excessive.

Theme 2: Weight gain is a source of concern for some: “Weight gain is something that’s always in my mind since I got pregnant”

About half of participants (n = 11) reported that their weight and weight gain during pregnancy were constantly on their minds. In addition, these participants reported worrying about how it would affect their health and that of their newborn. As previously mentioned, women who self-reported being a healthy weight (n = 10 out of 12) prior to pregnancy appeared most concerned about their gestational weight gain, and women who self-reported having obesity (n = 1 out of 1) and overweight (n = 4 out of 10) appeared most concerned about how their gestational weight gain could affect the health of their newborn.

Theme 3: Weight worries can wait: “Once the baby is born, I will lose the weight”

Despite being aware of their weight gain during pregnancy and worrying about it, about half of participants (n = 10) reported that they would worry about their weight status after having their baby. In addition, about two-third of participants (n = 15) reported being confident that with strong will, they would lose weight after childbirth. It is noteworthy that the majority of women (n = 11) reporting being confident loosing weight postpartum were those who self-reported being a healthy weight prior to pregnancy. For many women (n = 14), their confidence that they would be able to get back to their pre-pregnancy weight once the baby was born minimized their weight concerns or worrying too much about their weight gain.

Theme 4: Realization of weight gain: “Towards the end you just feel huge and all you want is to get back to your healthy weight”

The majority of participants (n = 19) stated that during the last few weeks of their pregnancy, their thoughts focused on having a safe delivery and on wanting to return to their pre-pregnancy weight. Overall, most participants (n = 17) expressed a sense of urgency about losing baby fat postpartum.

#### Interpersonal influences

Theme 5: Shared experience with and support from social networks: “Like my sister, when she was pregnant she really put on a lot of weight”

Many participants (n = 17) reported that family members and close friends gained a lot of weight during pregnancy, and this shared experience with their immediate social network made GWG more acceptable and led participants to think they also would gain more weight during pregnancy than what they viewed as being the right amount. Participants seemed to believe that they would also lose this extra pregnancy weight since family members and close friends who had gained weight during pregnancy were able to do so.

#### Organizational influences

Theme 6: Postpartum weight loss has not yet been discussed: “The focus now is on the pregnancy and on the baby”

When asked if they had discussed postpartum weight loss with their primary health care providers, the majority (n = 20) of participants reported that they had not but that they expected that would become the focus of their postpartum health care visits.

## Discussion

The findings of this exploratory qualitative study provide insight into sociocultural and interpersonal influences on first-time pregnant, low-income immigrant Latina women’s beliefs, attitudes, and experiences with GWG. This study’s findings indicate that participants were uncertain if their GWG was within a healthy range. Overall, findings showed that participants knew that GWG should be limited, but were not sure what the amount should be. Furthermore, the majority of participants reported attitudes of acceptance of and resignation to excessive GWG as being part of pregnancy. Several women appeared to believe that they did not have control over their weight gain during pregnancy. These findings concur with prior research conducted among Latina women [8,16–18,28] and have implications for the interventions designed for this population, a group at increased risk of excessive GWG and associated adverse consequences [9,15,16,18].

Women in the current study appeared to be more concerned about their baby’s health than about excessive GWG and their personal health. Women felt that that as long their baby was developing healthfully they did not yet need to worry about excessive GWG. This finding may reflect that participants in this study were not aware of the close link between a mother and child’s health and suggests the need for education about the short- and long-term consequences of excessive GWG for both mother and baby [9,10].

Women’s acceptance of GWG above a healthy range appeared to be shared and reinforced by their friends and family members. This finding is supported by prior research among Latina women revealing widespread sociocultural beliefs that reinforce that greater GWG is associated with healthier babies [3,4,28]. Combined, these studies indicate the need for increased awareness and education of the potential adverse consequences of excessive GWG for both mothers and babies. Furthermore, interventions designed to address GWG among Latina women should include women’s immediate social support networks of family members and friends [4,28].

Similar to previous studies, findings of the current study revealed that low-income Latina women perceived pregnancy as a time to set aside worries about eating habits [3,12,13,29]. Furthermore, as found in the current study, prior research shows that cultural beliefs enforce the idea that pregnancy is a unique time when women are allowed to give into cravings and eat for two [12,28]. Additionally, findings from the current study concur with previous research [12,28,29] and suggest that women’s social support networks influenced their sociocultural beliefs about weight gain during pregnancy. For example, nearly all women in this study reported that the social acceptance of the weight gain during pregnancy tended to alleviate the pressures they felt about what they perceived as the right amount of GWG. As mentioned above, these findings suggest that effective interventions aimed at prevention of excessive GWG among low-income Latina women should target not only pregnant Latina women but also their immediate social support networks [12,13,28,29].

## Limitations

Findings of the present study should be interpreted in light of some limitations. This was an exploratory study of first-time pregnant Latina women’s beliefs, attitudes, and experiences with GWG, and findings are based on a nonrandom, purposive, and relatively small sample of women living in MA and RI. Study participants may have been motivated to participate in the study due to heightened interest in the topic (GWG) since recruitment materials indicated the topic was a focus of the study. This study did not assess women’s physical activity levels and or perceptions of physical activity and diet, both of which are factors influencing GWG. Finally, this study relied on self-reported information from participants, including the self-reported BMI category, which may lead to misclassification (underestimation). Overweight women or women with obesity may be more likely to misclassify their weight status than women in a healthy weight range [30]. Finally, this study did not assess health care providers’ counseling practices, and prior studies suggest that women and health care providers report conflicting views on counseling [31].

Future research can address these limitations by examining perspectives from health care providers from these and other communities across the U.S. and comparing differences by women’s country of origin. Additionally, future studies focusing on Latina women should assess physical activity and diet using both objective and subjective assessment methods. Moreover, quantitative research that builds on the qualitative findings reported in the present study is needed on both pregnant Latina women and health care providers to quantify women’s beliefs, attitudes, and experiences with GWG.

## Conclusions

Latina women represent the largest portion of minority births and have had the highest birth rate in the U.S. for over 20 years. Therefore, understanding factors that influence pregnant Latina women’s beliefs, attitudes, and experiences with GWG is central to the prevention of excessive GWG and its related adverse health consequences for both mothers and their babies. Findings from the current study revealed that sociocultural and interpersonal factors influence the beliefs, attitudes, and experiences with GWG of low-income, immigrant Latina women pregnant with their first child. This information can be used to tailor prenatal care practices and interventions aimed at altering modifiable risk factors associated with excessive GWG.

## Supporting information

S1 Fig Consolidated criteria for reporting qualitative research. COREQ_Checklist_GWG-Latinas.
(PDF)
